# Robotic-arm assisted total knee arthroplasty has a learning curve of seven cases for integration into the surgical workflow but no learning curve effect for accuracy of implant positioning

**DOI:** 10.1007/s00167-018-5138-5

**Published:** 2018-09-17

**Authors:** Babar Kayani, S. Konan, S. S. Huq, J. Tahmassebi, F. S. Haddad

**Affiliations:** 1 University College Hospital, 235 Euston Road, Bloomsbury, London, NW1 2BU UK; 2 Princess Grace Hospital, 42-52 Nottingham Place, Marylebone, London, W1U 5NY UK

**Keywords:** Implant positioning, Learning curve, Operative time, Robotics, TKA, Total knee arthroplasty, Total knee replacement

## Abstract

**Purpose:**

The primary objective of this study was to determine the surgical team’s learning curve for robotic-arm assisted TKA through assessments of operative times, surgical team comfort levels, accuracy of implant positioning, limb alignment, and postoperative complications. Secondary objectives were to compare accuracy of implant positioning and limb alignment in conventional jig-based TKA versus robotic-arm assisted TKA.

**Methods:**

This prospective cohort study included 60 consecutive conventional jig-based TKAs followed by 60 consecutive robotic-arm assisted TKAs performed by a single surgeon. Independent observers recorded surrogate markers of the learning curve including operative times, stress levels amongst the surgical team using the state-trait anxiety inventory (STAI) questionnaire, accuracy of implant positioning, limb alignment, and complications within 30 days of surgery. Cumulative summation (CUSUM) analyses were used to assess learning curves for operative time and STAI scores in robotic TKA.

**Results:**

Robotic-arm assisted TKA was associated with a learning curve of seven cases for operative times (*p* = 0.01) and surgical team anxiety levels (*p* = 0.02). Cumulative robotic experience did not affect accuracy of implant positioning (n.s.) limb alignment (n.s.) posterior condylar offset ratio (n.s.) posterior tibial slope (n.s.) and joint line restoration (n.s.). Robotic TKA improved accuracy of implant positioning (*p* < 0.001) and limb alignment (*p* < 0.001) with no additional risk of postoperative complications compared to conventional manual TKA.

**Conclusion:**

Implementation of robotic-arm assisted TKA led to increased operative times and heightened levels of anxiety amongst the surgical team for the initial seven cases but there was no learning curve for achieving the planned implant positioning. Robotic-arm assisted TKA improved accuracy of implant positioning and limb alignment compared to conventional jig-based TKA. The findings of this study will enable clinicians and healthcare professionals to better understand the impact of implementing robotic TKA on the surgical workflow, assist the safe integration of this procedure into surgical practice, and facilitate theatre planning and scheduling of operative cases during the learning phase.

**Level of evidence:**

II.

## Introduction

Total knee arthroplasty (TKA) is an established and cost-effective treatment for patients with symptomatic end-stage knee osteoarthritis [7]. However, recent studies have shown that 20% of patients still remain dissatisfied following TKA [1, 18]. Accuracy of implant positioning and limb alignment are important prognostic factors that influence patient satisfaction, clinical outcomes, and long-term implant survivorship following TKA [8, 17, 18, 22]. Evolution in surgical technology has led to the development of robotic-arm assisted TKA, which uses a preoperative computerised tomography (CT) scan to create a patient-specific computer-aided design (CAD) model of the patient’s unique knee anatomy. The surgeon is able to virtually select the desired implant position and alignment, and an intraoperative robotic arm helps to execute this plan with a high degree of accuracy [11, 12]. Robotic-arm assisted TKA improves the accuracy of bone resection, reduces outliers in postoperative limb alignment, and decreases iatrogenic bone and periarticular soft tissue injury compared to conventional manual TKA [11, 12, 21, 22].

Existing studies on the learning curve of robotic-arm assisted TKA have used operative times as exclusive markers of surgical competence, and found surgical proficiency may be achieved by high-volume arthroplasty surgeons within a few months [4, 20]. It is possible to improve on these existing studies by comparing a more comprehensive range of learning outcome measures including operative times of individual stages of the robotic procedure, surgical team comfort levels, accuracy of implant positioning, restoration of limb alignment, and postoperative complications. In this study, cumulative summation (CUSUM) analyses will be used to assess incremental changes in these study outcomes during progression of the robotic TKA learning curve [13], and the findings compared to baseline values from a cohort of patients undergoing conventional manual TKA by the same operating surgeon. This data will be used to ascertain inflexion points at which the surgeon transitions from the learning phase to the proficiency phase in more detail. The findings of this study will enable clinicians and healthcare professionals to better understand the impact of implementing robotic TKA on the surgical workflow, facilitate theatre planning and scheduling of operative cases, and understand any additional risks or complications during the acquisition of surgical proficiency.

The primary objective of this study was to determine the surgical team’s learning curve for robotic-arm assisted TKA through assessments of operative times, surgical team comfort levels, accuracy of implant positioning, limb alignment, and postoperative complications. The hypothesis was that cumulative experience with robotic-arm assisted TKA would lead to improved operative times and surgical team comfort levels but there would be no learning effect for accuracy of implant positioning or limb alignment. The secondary objectives were to compare accuracy of implant positioning and limb alignment in patients undergoing robotic-arm assisted TKA versus conventional jig-based TKA.

## Materials and methods

This prospective cohort study included 120 patients with symptomatic knee osteoarthritis undergoing primary TKA between 2016 and 2017. This included 60 consecutive patients undergoing conventional jig-based TKA followed by 60 consecutive patients receiving robotic-arm assisted TKA. Patients were allocated to their treatment group based on the date of their surgery relative to installation of the robotic device into the study institution. Conventional jig-based TKA was performed prior to installation of the robotic device, and robotic-arm assisted TKA performed after its installation. Patients were not randomized but this enabled assessment of learning curves associated with complete transition from conventional jig-based TKA to robotic-arm assisted TKA. All operative procedures were performed by the senior author who is experienced in performing conventional jig-based TKA and had undergone cadaveric training on robotic-arm assisted TKA. The robotic group was the first cohort of patients undergoing robotic-arm assisted TKA under the operating surgeon.

Inclusion criteria for this study included the following: Patients with knee osteoarthritis undergoing primary total knee arthroplasty; patients between 18 and 80 years of age; surgery undertaken using the conventional jig-based or robotic-arm assisted technique; surgery performed by the senior author. Exclusion criteria included the following: conversion of unicompartmental knee arthroplasty to TKA (*n* = 6); prior infection of knee joint (*n* = 1); arthroplasty for fracture or previous osteotomy (*n* = 2); and underlying neurological dysfunction compromising mobility (*n* = 1). Patients undergoing conventional jig-based TKA and robotic-arm assisted TKA were well matched for baseline characteristics (Table 1). In both treatment groups, the standard medial parapatellar approach was used with implantation of the cemented Stryker Triathlon (Stryker Navigation, Kalamazoo, Michigan, USA), cruciate substituting knee system and asymmetrical patella resurfacing. Two independent observers collected all study outcomes and both were blinded to each other’s recordings. These observers were not involved in the surgical planning, operative procedure, or postoperative treatment process. Written informed consent was obtained from all study participants.

**Table 1:** Baseline characteristics in patients undergoing conventional jig-based TKA versus robotic-arm assisted TKA

Characteristic	Conventional jig-based TKA (*n* − 60)	Robotic-arm assisted TKA (*N* = 60)	*p* value
Age (years)	68.7 ± 6.1	67.6 ± 7.6	n.s.
Body mass index (kg/m^2^)	26.1 ± 3.6	27.2 ± 3.6	n.s.
Gender (female/male)	F 33 (55.0%)	F 32 (53.3%)	n.s.
	M 27 (45.0%)	M 28 (46.7%)	
ASA grade	I—24 (40.0%)	I—21 (35.0%)	
	II—32 (53.7%)	II—33 (55.0%)	n.s.
	III—4 (6.7%)	III—6 (10.0%)	
Side intervention (right/left)	R 29 (48.3%)	R 33 (55.0%)	n.s.
	L 31 (51.7%)	L 27 (45.0%)	

Summary statistics are: *N* (percentage) or mean with standard deviation

*BMI* body mass index, *ASA score* American Society of Anaesthesiologists score

All patients underwent routine preoperative anteroposterior weight-bearing knee radiographs, lateral knee radiographs, and full-length hip-to-ankle weight-bearing radiographs. In both treatment groups, the operating surgeon used Traumacad software (Traumacad, Petach-Tikva, Israel) with plain radiographs to preoperatively template implant sizes and positions. Full-length hip-to-ankle radiographs were used to guide femoral and tibial resection angles to achieve neutral mechanical alignment. Lateral knee radiograph was used to select the femoral component size and position to restore the patient’s native posterior condylar offset ratio whilst avoiding overhang or notching of the femur. Tibial implant position and size were selected to restore native posterior tibial slope and avoid any anteroposterior overhang. On the anteroposterior knee radiograph, femoral and tibial implant positons and sizes were selected to achieve maximum mediolateral contact whilst avoiding overhang. In patients undergoing robotic-arm assisted TKA, preoperative CT scan and CAD model were used to select the optimal implant positioning and implant sizes for achieving the desired bone coverage and limb alignment.

### Surgical technique

Conventional jig-based TKA was performed using standard instrumentation with alignment jigs to guide bone resection. Extramedullary referencing was used to perform tibial bone resection perpendicular to the mechanical axis of the tibia in the coronal plane with the aim of matching anatomical anteroposterior slope in the sagittal plane. The femur was prepared using an intramedullary alignment jig with the distal cutting block positioned so that the distal femoral cut was at 5°–7° valgus angle depending on the pre-existing deformity. The distal femoral cutting block was positioned in 3° or greater of external rotation using the transepicondylar axis. Flexion and extension gaps were checked and appropriate soft tissue releases performed to ensure the knee was balanced. No further intraoperative adjustments or tailoring of implant positioning were performed to account for individual patient anatomy.

In patients undergo robotic-arm assisted TKA, distal femoral and proximal tibial bicortical registration pins were inserted and fixed arrays mounted onto these to enable intraoperative dynamic referencing. Bone registration was performed by intraoperatively mapping radiological landmarks displayed on the computer screen to verify anatomy and establish bone geometry. Joint balancing captured femoral and tibial poses with corrective forces, assessed kinematics through the arc of motion, and enabled fine tuning of implant positioning based on laxity of the soft tissue envelope. An intraoperative surgeon-controlled robotic arm with visual, tactile, and audio feedback was then used to execute the preoperative plan to within 2 mm of the planned bone resection. Tibial and femoral osteotomies in the coronal plane were performed perpendicular to the tibial and femoral mechanical axes, respectively, to achieve neutral overall alignment. In the sagittal plane, 0°–5° of femoral component flexion were used to optimise implant sizing whilst preventing notching. The tibial slope was initially set to zero degrees and then adjusted as required based on intraoperative assessment of the flexion gap and range of motion. Optical motion capture technology was used to assess limb alignment, range of motion, flexion and extension gaps, and arc of motion with trial implants prior to definitive selection and cement implantation of final components.

### Outcome measures

#### Interclass correlation coefficient

All radiological measurements were recorded by each observer at 28 days apart and findings compared to assess for intra-observer agreement. Radiological measurements were compared between the two observers to assess for inter-observer agreement. Interclass correlation coefficient was 0.9 (95% CI 0.8–1.0) for intra-observer agreement and 0.9 (95% CI 0.8–0.9) for inter-observer agreement in all study outcomes, which indicated good agreement on all radiological parameters assessed by the two independent observers.

#### Operative time

Operative time was defined as time from initial surgical incision to final wound closure. In robotic-arm assisted TKA, surgical times for the following parts of the procedure were recorded: Set up of surgical tray, robotic device, and instruments; surgical approach and insertion of registration pins; bone registration; joint balancing; bone preparation; implant trialling; cement implantation of final prosthesis; and overall operative time.

#### Surgical team anxiety levels

The Spielberger State-Trait Anxiety Inventory (STAI) questionnaire is a validated subjective assessment tool for quantifying an individual’s stress levels with individual traits arising from the clinical environment [14]. The six-item questionnaire has a 4-point rating scale and total scores range from 6 to 24, with higher values indicating higher levels of stress. The STAI questionnaire was completed by each member of the surgical team prior to the surgical time-out in all study patients. The surgical team included the operating surgeon, two consultant anaesthetists, two senior scrub nurses, one operating department practitioner (ODP), and one circulating nurse.

#### Implant positioning and limb alignment

All patients underwent postoperative anteroposterior weight-bearing and lateral knee radiographs, and full-length hip-to-ankle weight-bearing radiographs. Accuracy of implant positioning and limb alignment were assessed by comparing the values achieved in the postoperative radiographs to the planned values in the corresponding preoperative radiographs. Femoral and tibial axes were used as reference markers as described by Bell et al. [2]. Accuracy of achieving the planned femoral and tibial implant positioning were assessed using the techniques described by Moon et al. [15]. The femoral coronal implant alignment was measured as the medial angle subtended by the femoral mechanical axis and the line connecting the distal points of the medial and lateral condyles of the femoral component. The femoral sagittal implant alignment was calculated as the angle subtended between the perpendicular line running proximally from the distal femoral surface in contact with the femoral component and the femoral mechanical axis. The tibial coronal implant alignment was measured as the medial angle subtended by the tibial mechanical axis and the medial to lateral axis of the tibial implant. The tibial sagittal alignment was calculated as the angle between the tibial mechanical axis and anterior to posterior axis of the tibial implant. Anteroposterior plain knee radiographs were used to measure the joint line height by calculating the perpendicular distance from a line extending through the distal points of the femoral condyles and a parallel line extending to the fibular head. True lateral knee radiographs were used to calculate the posterior tibial slope and posterior condylar offset ratio (PCOR) using the methods described by Gaudiani et al. [6] and Johal et al. [9] respectively.

#### Complications

All patients were reviewed in outpatient clinic at 30 days following surgery by the independent observers for clinical assessment and full weight-bearing radiographs performed. Any postoperative complications and their respective treatments during this follow-up period were recorded for analysis.

Hospital review board approval was acquired from the host institution (Reference: 241413, Princess Grace Hospital, 42–52 Nottingham place, Marylebone, London, W1U 5NY, UK) before commencement of the study. Further Research Ethics Committee (REC) or Health Research Authority (HRA) approval was not required for this study.

### Statistical analysis

Sample size calculation was performed using operative time as the primary outcome measure and published data on operative times with similar surgical techniques for TKA. The minimal clinical difference was set at 5 min and standard deviation at 10 min [19]. This study required 60 patients in each arm to detect this minimum difference in operative time using a two-tailed, two-sample *t* tests with a power of 80% and significance level of 5%. Due to the limited follow-up time, no further adjustments were made to the sample size calculation to account for sample size attrition during follow-up.

The CUSUM sequential analysis tool was used to assess learning curves in robotic-arm assisted TKA for operative time and surgical team stress levels as assessed using the STAI questionnaire. Standardised target values for the CUSUM analyses were set using the overall mean values for these outcome measures from the robotic-arm assisted TKA group. CUSUM values represent a running total of the differences between the value of each data point and the standardised target values for each outcome. Learning curves for accuracy of implant position and limb alignment in robotic-arm assisted TKA were assessed by calculating root mean square error values for radiological outcomes and assessing progression in groups of ten patients. Categorical data were compared using the chi square test and Fisher’s exact test where greater than 25% of cells had less than five cases. Normally distributed continuous variables were compared using independent *t* tests for unpaired variables, paired *t* test for paired (matched) variables, and one-way ANOVA for multiple variables, The Mann–Whitney test was used for non-parametric data. Statistical significance was set at *p* < 0.05 for all statistical tests. All statistical analyses were performed using SPSS software version 21 (SPSS Inc., Chicago, IL, USA).

## Results

### Operative times

**Fig. 1:**
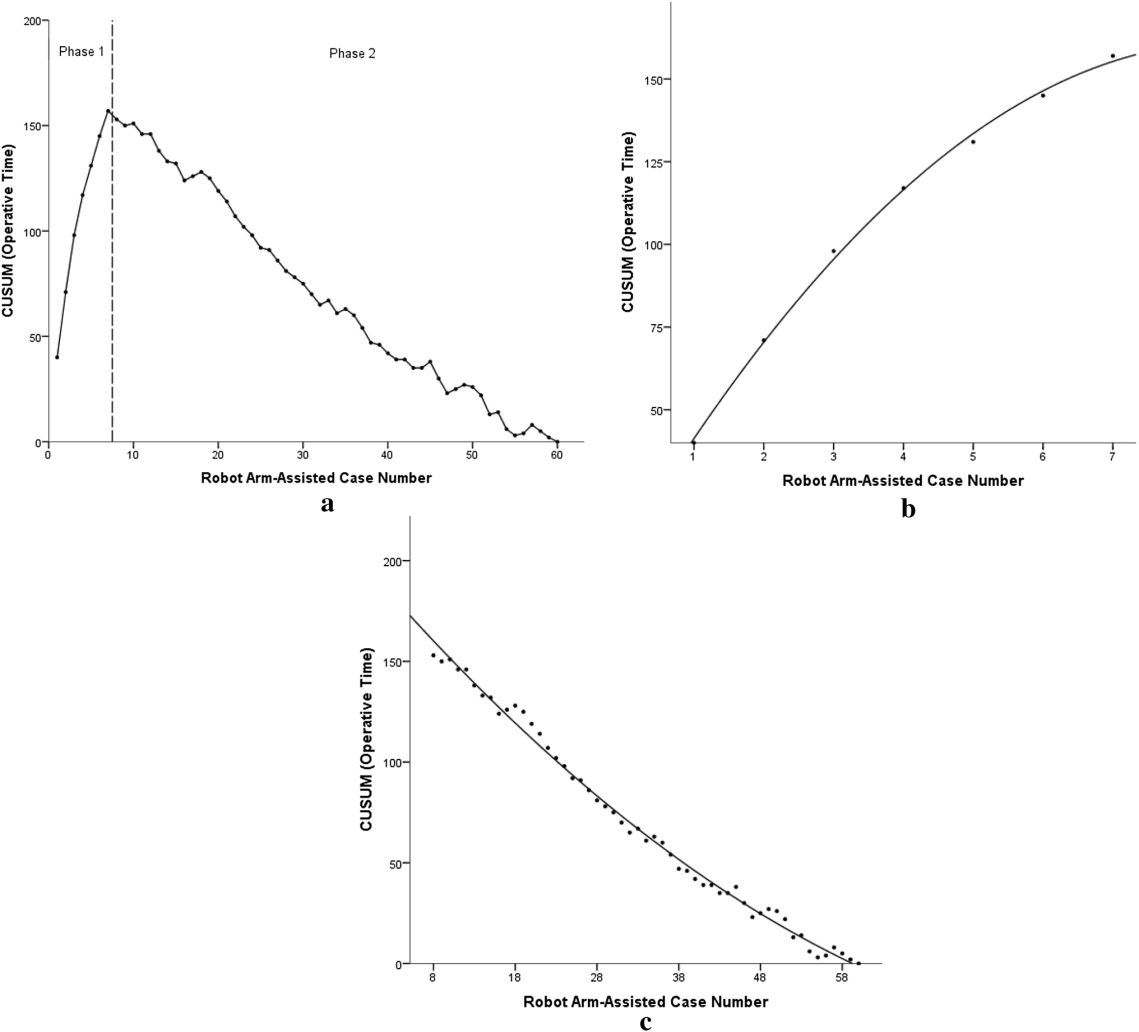
CUSUM analysis charts demonstrating the learning curve for operative time in patients undergoing robotic-arm assisted TKA. **a** CUSUM chart for operative times in consecutive robotic-arm assisted TKA cases. Dashed vertical line represents the inflexion point at which the learning curve transitions from the learning phase (phase 1) to the proficiency phase (phase 2). **b** CUSUM chart for phase 1 robotic-arm assisted TKA cases. **c** CUSUM chart for phase 2 robotic-arm assisted TKA cases

In robotic-arm assisted TKAs, CUSUM analysis for operative times revealed a sharp inflexion point after the initial seven cases, which helped to identify two distinct phases in the learning curve (Fig. 1). Phase 1 represents the initial learning segment and phase 2 represents the proficiency stage in robotic-arm assisted TKA. Comparison of the two phases demonstrated phase 1 procedures to be significantly longer (*p* = 0.01) with no differences in baseline characteristics compared to phase 2 (Tables 2, 3).

**Table 2:** Operative data in patients undergoing robotic-arm assisted TKA

Operative stage (mins)	Cases 1–10	Cases 11–20	Cases 21–30	Cases 31–40	Cases 41–50	Cases 51–60	*p* value
Instrument, robotic device, and surgical tray set-up	14.8 ± 4.3	8.7 ± 1.9	8.9 ± 1.2	9.1 ± 1.7	8.8 ± 1.3	9.2 ± 1.5	0.01*
Surgical approach	7.8 ± 1.5	7.5 ± 1.9	7.2 ± 1.6	7.0 ± 1.2	7.5 ± 1.1	7.2 ± 1.8	n.s.
Bone registration	15.8 ± 4.1	11.1 ± 1.2	10.6 ± 1.8	10.9 ± 1.4	11.5 ± 1.8	11.1 ± 1.6	0.02*
Joint balancing	14.3 ± 3.8	8.9 ± 1.2	8.7 ± 0.9	8.8 ± 1.2	9.1 ± 1.5	9.0 ± 1.8	0.03*
Bone preparation	16.2 ± 3.4	11.9 ± 1.2	11.7 ± 1.6	11.9 ± 1.8	10.7 ± 1.2	11.8 ± 1.6	0.03*
Implant Trialling	7.8 ± 1.1	7.6 ± 1.9	7.7 ± 1.5	7.9 ± 1.2	7.1 ± 1.1	8.5 ± 1.5	n.s.
Cement implantation	14.6 ± 1.2	14.2 ± 1.4	13.6 ± 1.9	14.1 ± 1.3	13.8 ± 1.4	13.7 ± 1.3	n.s.
Closure	6.5 ± 1.5	5.8 ± 1.4	5.8 ± 1.1	5.9 ± 0.7	6.1 ± 1.2	5.8 ± 0.9	n.s.
Overall operating time	83.1 ± 10.5	67.2 ± 4.1	65.3 ± 3.2	66.1 ± 3.9	67.1 ± 3.7	67.2 ± 4.3	0.01*

Summary statistics are: mean value and standard deviation. *p* value for trend

*Statistically significant fall in study outcome after cases 1–10

**Table 3:** Comparison of learning curve phases in patients undergoing robotic-arm assisted TKA

Characteristic	Phase 1 (*n* = 7)	Phase 2 (*n* = 53)	*p* value
Age (years)	66.9 ± 6.9	69.1 ± 7.6	n.s.
BMI (kg/m^2^)	25.3 ± 4.4	27.4 ± 3.4	n.s.
ASA grade 3	1 (14.2%)	5 (9.4%)	n.s.
Male gender	3 (42.9%)	25 (47.2%)	n.s.
Preoperative Hb (g/L)	132.4 ± 5.8	133.6 ± 9.8	n.s.
Postoperative Hb change (g/L)	15.1 ± 7.6	15.7 ± 6.9	n.s.
Operative time (min)	89.2 ± 4.2	66.8 ± 3.5	0.01

Summary statistics are: *N* (percentage) or mean value and standard deviation

*BMI* body mass index, *ASA score* American Society of Anaesthesiologists score, *HB* haemoglobin

### Surgical team anxiety levels

**Fig. 2:**
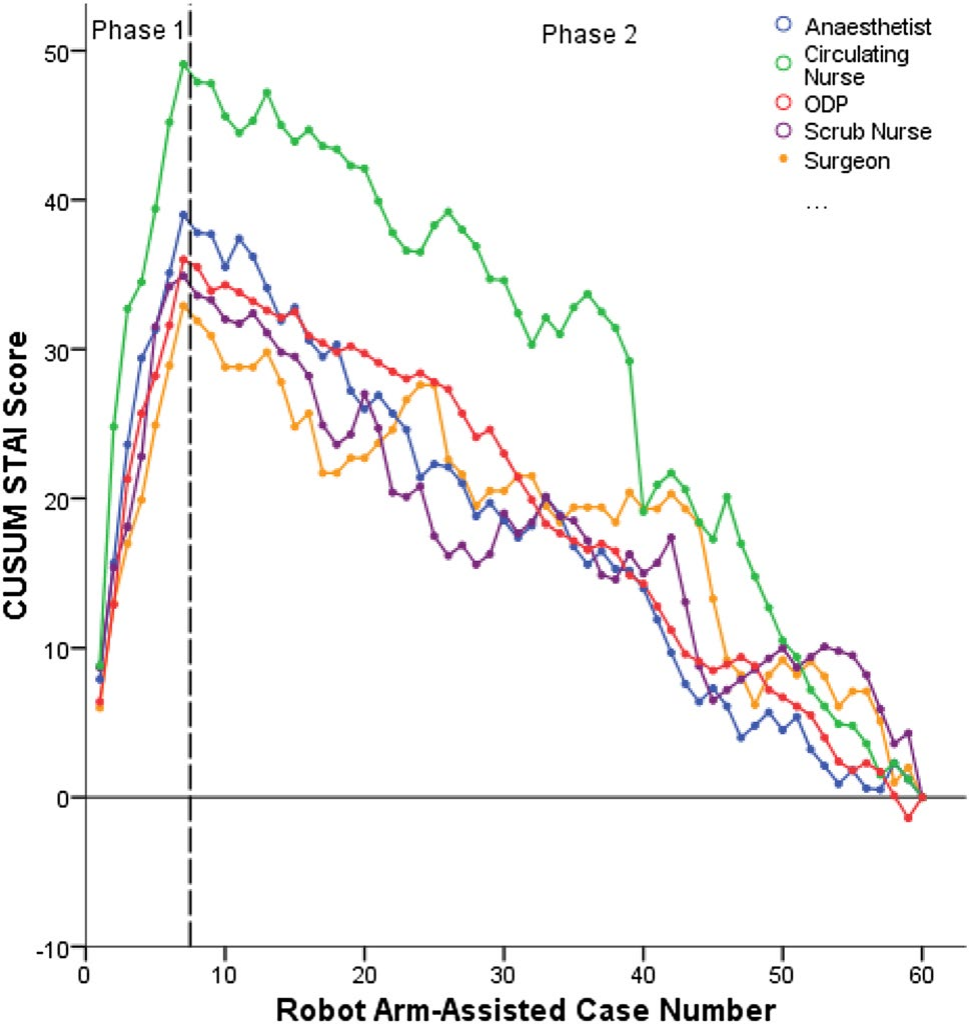
Chart displaying CUSUM analysis for STAI scores amongst all surgical team members in robotic-arm assisted TKA

**Fig. 3:**
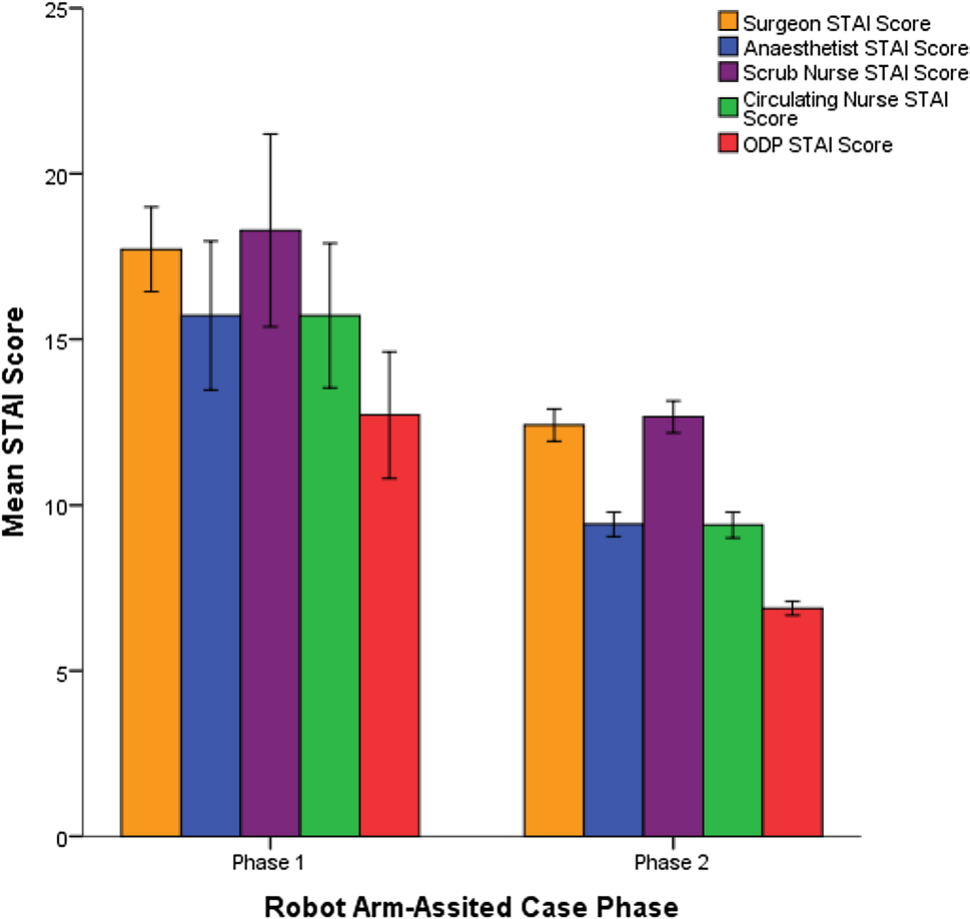
Chart comparing STAI scores between learning phases for all members of the surgical team in robotic-arm assisted TKA

CUSUM analysis of preoperative stress levels as assessed using the STAI questionnaire revealed an inflexion point after seven robotic cases (*p* = 0.02) in a pattern similar to operative times in robotic-arm assisted TKA (Fig. 2). Further analysis revealed STAI scores to be significantly higher in phase 1 than in phase 2 for all members of the surgical team (Fig. 3).

### Implant positioning and limb alignment

**Fig. 4:**
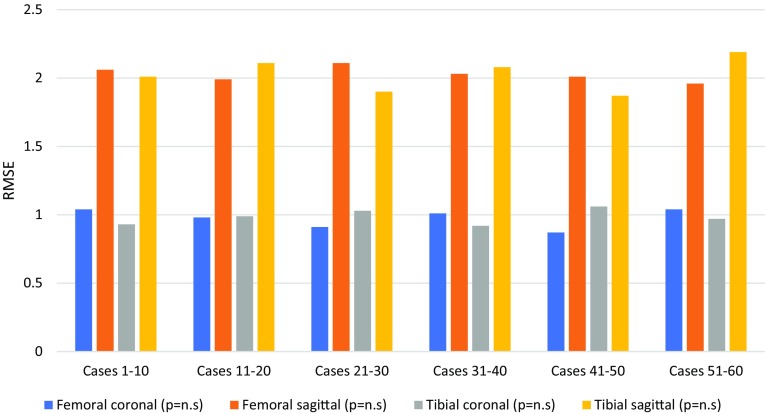
Bar chart showing changes in root mean square error (RMSE) for accuracy in femoral and tibial implant positioning (degrees) in consecutive patient groups undergoing robotic-arm assisted TKA

**Fig. 5:**
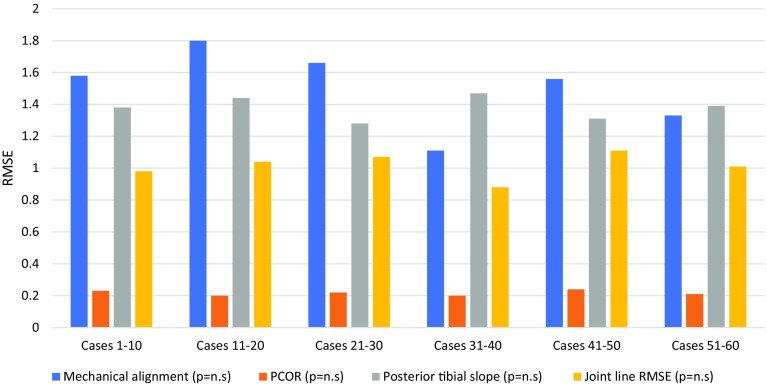
Bar chart showing changes in root mean square error (RMSE) for accuracy in achieving planned mechanical alignment (degree), posterior condylar offset ratio (PCOR), posterior tibial slope (degrees), and joint line restoration (mm) in consecutive patient groups undergoing robotic-arm assisted TKA

There was no learning curve effect of robotic-arm assisted TKA on accuracy of achieving the planned implant position and limb alignment (Table 4; Figs. 4, 5). Robotic-arm assisted TKA improved accuracy in achieving the planned implant positions compared to conventional jig-based TKA (Table 5).

**Table 4:** Accuracy of implant positioning and limb alignment in patients undergoing robotic-arm assisted TKA

Radiological outcome	Cases 1–10	Cases 11–20	Cases 21–30	Cases 31–40	Cases 41–50	Cases 51–60	*p* value
Mechanical alignment RMSE (degrees)	1.6 ± 0.8	1.8 ± 1.0	1.7 ± 1.2	1.1 ± 0.6	1.6 ± 0.9	1.3 ± 1.0	n.s.
PCOR RMSE	0.2 ± 0.1	0.2 ± 0.1	0.2 ± 0.1	0.2 ± 0.1	0.2 ± 0.1	0.2 ± 0.1	n.s.
Posterior tibial slope RMSE (degrees)	1.4 ± 0.7	1.4 ± 0.9	1.3 ± 0.6	1.5 ± 0.7	1.3 ± 0.6	1.4 ± 0.7	n.s.
Joint line RMSE (mm)	1.0 ± 0.4	1.0 ± 0.6	1.1 ± 0.6	0.9 ± 0.6	1.1 ± 0.7	1.0 ± 0.6	n.s.
Femoral coronal RMSE (degrees)	1.0 ± 0.4	1.0 ± 0.3	0.9 ± 0.4	1.0 ± 0.4	0.9 ± 0.5	1.0 ± 0.4	n.s.
Femoral sagittal RMSE (degrees)	2.1 ± 0.8	2.0 ± 0.7	2.1 ± 0.5	2.0 ± 0.5	2.0 ± 1.0	1.9 ± 0.5	n.s.
Tibial coronal RMSE (degrees)	0.9 ± 0.3	1.0 ± 0.5	1.0 ± 0.7	0.9 ± 0.5	1.1 ± 0.4	1.0 ± 0.5	n.s.
Tibial sagittal RMSE (degrees)	2.0 ± 0.5	2.1 ± 0.5	1.9 ± 0.7	2.1 ± 0.7	1.9 ± 0.8	2.2 ± 0.5	n.s.

Summary statistics are: RMSE (root mean square error) with standard deviation

**Table 5:** Study outcomes in patients undergoing conventional jig-based TKA versus robotic-arm assisted TKA

Characteristic	Conventional jig-based TKA (*n* = 60)	Robotic-arm assisted TKA (*n* = 60)	*p* value
Operative time (mins)	62.1 ± 5.7	69.4 ± 8.1	n.s.
Preoperative STAI score			
Operating surgeon	12.1 ± 3.4	13.0 ± 4.1	n.s.
Anaesthetist	9.1 ± 2.5	9.7 ± 2.5	n.s.
Scrub nurse	12.8 ± 3.1	13.3 ± 2.6	n.s.
Circulating nurse	11.1 ± 2.1	10.2 ± 2.9	n.s.
ODP	8.6 ± 3.1	7.6 ± 2.4	n.s.
Postoperative radiological outcomes			
Mechanical alignment RMSE (degrees)	3.2 ± 1.2	1.5 ± 0.9	< 0.001*
PCOR	0.3 ± 0.1	0.2 ± 0.1	n.s.
Posterior tibial slope RMSE (degrees)	3.4 ± 1.1	1.4 ± 0.6	< 0.001*
Joint line RMSE (mm)	2.9 ± 1.4	1.0 ± 0.6	< 0.001*
Femoral coronal alignment RMSE (degrees)	4.1 ± 1.1	1.0 ± 0.4	< 0.001*
Femoral sagittal alignment RMSE (degrees)	4.2 ± 0.8	2.1 ± 0.7	< 0.001*
Tibial coronal alignment RMSE (degrees)	3.6 ± 0.8	1.0 ± 0.5	< 0.001*
Tibial sagittal alignment RMSE (degrees)	3.9 ± 1.0	2.0 ± 0.6	< 0.001*

Summary statistics are: mean with standard deviation

### Complications

There were two inpatient complications in this study, which included one patient from each treatment group. In conventional jig-based TKA, one patient had minor wound dehiscence from the distal part of the midline incision, which was treated with adhesive skin strips to approximate the wound edges and prophylactic antibiotics. In the robotic-arm assisted TKA group, one patient had minor wound dehiscence over the incision for the proximal tibial registration pins. This was treated with regular dressings and prophylactic oral antibiotics. Both patients made a satisfactory recovery with no further complications.

## Discussion

The most pertinent findings from this study are that robotic-arm assisted TKA was associated with a learning curve of seven cases for operative times and surgical team comfort levels but there was no learning curve for accuracy of implant positioning, limb alignment, posterior condylar offset ratio, posterior tibial slope, and joint line preservation. Robotic-arm assisted TKA was associated with improved accuracy of implant positioning and limb alignment with no additional risk of complications compared to conventional jig-based TKA.

The operative time in robotic-arm assisted TKA progressively decreased over the initial seven cases as the surgical team became increasingly familiar with robotic technology and accustomed to the stages of robotic TKA. Most marked time improvements occurred with bone registration and operative times for this stage of the procedure decreased by over 50% during the initial learning phase. Intraoperative anatomical landmarks for bone registration were similar in all patients, and therefore, with increasing surgical experience, the surgeon was able to predict and pre-emptively place the bovie tip over the appropriate bone landmark for registration. More moderate improvements were observed in time for bone resection as the surgeon became progressively more responsive to feedback from the saw blade. As the surgeon became more adept with fine movements of the robotic arm and more receptive to the audio, visual, and tactile feedback, he was able to better control the movements of the arm and preform bone cuts with greater efficiency. This study also found that mean time for joint balancing during the proficiency stage was 8.9 min (range 7–12 min). During this stage, the surgeon assessed knee kinematics through the arc of motion, flexion and extension gaps, range of movement, and stability using optical motion technology. Using this intraoperative data, the surgeon was able to fine-tune femoral and tibial bone resections to balance flexion and extension gaps without having to perform more extensive soft tissue releases as may often be required in conventional jig-based TKA [7, 8]. This may have helped to limit the overall observed difference in operative times between conventional TKA versus robotic TKA.

The findings of this study complement those of Sodhi et al. [20] that explored the learning curve of robotic TKA using operative time as an exclusive marker of surgical proficiency. The authors reviewed operative times in two different surgeons and found mean operative times in the first 20 robotic cases were increased compared to each surgeon’s mean operative time for conventional jig-based TKA [20]. Operative times for the initial 20 robotic TKA cases ranged from 71 to 104 min for surgeon 1, and ranged from 74 to 142 min in surgeon 2. The wide variation in operative times over the first 20 cases suggest that the learning curve may have already been in effect and operative times comparable to those of conventional TKA may have been observed much earlier than reported. The authors also reported that after the initial learning phase, operative times in robotic-arm assisted TKA were comparable to those of conventional jig-based TKA, which is consistent with the findings of this study. In theory, robotic-arm assisted surgery helps to produce a more streamlined surgical procedure by reducing the need for instrument trays, alignment guides, and cutting blocks, enabling more rapid computer-guided bone resections, and reducing need for trialling due to the high accuracy of preoperative surgical planning [21]. However, in this study, these potential benefits with robotic TKA did not translate to faster operative times compared to conventional jig-based TKA.

Implementation of robotic-arm assisted TKA was associated with heightened levels of anxiety amongst the surgical team during the initial learning phase. This is important as higher levels of stress and mental strain are associated with diminished operative performance, poor decision-making, and reduced technical skills [14]. In this study, improvements in the surgical team’s anxiety levels with robotic-arm assisted TKA followed in a trend similar to that of operative times with baseline STAI scores reached after seven cases. Progressive improvements in anxiety scores during this initial learning phase correlated with the surgical team becoming more proficient with setting up the new trays and instruments, positioning the robotic machine in theatre, attaching the burr to the robotic arm, and proactively preparing the registration pins, check points, and arrays. As the team became more confident with these steps, subjective anxiety levels and operative times diminished. The highest anxiety levels were observed in the operating surgeon and scrub nurse during the initial learning phase but these did not translate into any differences in accuracy of implant positioning or limb alignment.

Cumulative robotic experience did not impact the accuracy of achieving the planned implant positioning, limb alignment, posterior condylar offset ratio, posterior tibial slope, or native joint line restoration. Robotic-arm assisted TKA uses bone registration to confirm intraoperative spatial orientation of the limb and fixed arrays accurately track the femoral and tibial bone resection windows throughout the procedure. Stereotactic boundaries also confine bone resection to the limits of the haptic windows, which helps to reduce manual errors in bone resection and iatrogenic soft tissue injury from the handheld sawblade used in conventional TKA [11, 12]. The robotic procedure, therefore, limits bone resection to the preoperative surgical plan and this may have helped to limit any surgeon-induced errors in implant positioning during the learning phase.

Robotic-arm assisted TKA was associated with improved accuracy in implant positioning and limb alignment compared to conventional jig-based TKA, which is important as these outcomes affect functional recovery, clinical outcomes, and long-term implant survivorship [8, 18, 19, 22]. The findings of this study are consistent with those of Song et al. who performed a prospective randomized study on 100 patients undergoing primary TKA and found robotic-arm assisted surgery improved accuracy of mechanical alignment with reduced outliers of greater than 3° in planned alignment compared to conventional manual TKA (0 versus 24%, *p* < 0.001) [22]. Bellemans et al. reviewed outcomes in 25 patients undergoing robotic-arm assisted TKA  and found femoral and tibial implant alignment within 1° of the planned positions in all three planes [3]. Improved accuracy in preserving the native posterior tibial slope and joint line within the robotic group in this study are also significant findings as previous studies have shown that these radiological outcomes correlate with improved patient satisfaction, stability, and kinematics through the arc of motion following TKA [5, 10].

There are several limitations of this study that must be appreciated when interpreting the findings. First, accuracy of implant positioning and limb alignment was measured using plain radiographs, which are not as accurate as CT scans. Second, different preoperative planning techniques were used in each treatment group, which may have affected the accuracy of implant positioning achieved with each treatment technique. Third, the surgical team in this study are all experienced in working with both conventional and navigated TKA in a high-volume arthroplasty centre, and therefore, their learning curve may not be directly transferrable to other less experienced teams. Fourth, follow-up time was limited to 30 days following surgery and so long-term data on functional outcomes, implant survivorship and revision rates were not available. Fifth, additional costs and impact on other operative cases due to increased operating times during the learning phase were not assessed.

The findings of this study will enable healthcare professionals to better understand the impact of implementing robotic-arm assisted TKA on the surgical workflow. Theatre planning and scheduling of operative cases should consider increased operative times and heightened levels of anxiety amongst the surgical team during this initial learning phase. As team members become more familiar and adept with robotic technology, comfort levels improve and theatre efficiency increases thereafter. After the initial learning phase of robotic-arm assisted TKA, operative times with robotic TKA will be comparable to those with conventional manual TKA. There is no impact of cumulative experience with robotic-arm assisted TKA on accuracy of implant positioning or limb alignment, which is important for the safe implementation of this procedure into routine surgical practice. Robotic-arm assisted TKA improves accuracy of implant positioning with no additional risk of postoperative complications at short-term follow-up compared to conventional manual TKA.

## Conclusion

Implementation of robotic-arm assisted TKA alters the surgical workflow with increased operative times and heightened levels of anxiety amongst the surgical team for the initial seven cases but this does not translate to any compromise in the accuracy of implant positioning. Robotic-arm assisted TKA improved accuracy of implant positioning and limb alignment compared to conventional jig-based TKA.
